# The genome sequence of the Red-clover Case-bearer, *Coleophora deauratella *Zeller, 1846

**DOI:** 10.12688/wellcomeopenres.22581.1

**Published:** 2024-07-10

**Authors:** Liam M. Crowley, Denise C. Wawman, University of Oxford and Wytham Woods Genome Acquisition Lab, Darwin Tree of Life Barcoding collective, Wellcome Sanger Institute Tree of Life Management, Samples and Laboratory team, Wellcome Sanger Institute Scientific Operations: Sequencing Operations, Wellcome Sanger Institute Tree of Life Core Informatics team, Tree of Life Core Informatics collective, Darwin Tree of Life Consortium

**Affiliations:** 1 Department of Biology, University of Oxford, Oxford, England, UK

**Keywords:** Coleophora deauratella, Red-clover Case-bearer, genome sequence, chromosomal, Lepidoptera

## Abstract

We present a genome assembly from an individual female *Coleophora deauratella* (the Red-clover Case-bearer; Arthropoda; Insecta; Lepidoptera; Coleophoridae). The genome sequence is 518.4 megabases in span. Most of the assembly is scaffolded into 31 chromosomal pseudomolecules, including the Z and W sex chromosomes. The mitochondrial genome has also been assembled and is 15.76 kilobases in length.

## Species taxonomy

Eukaryota; Opisthokonta; Metazoa; Eumetazoa; Bilateria; Protostomia; Ecdysozoa; Panarthropoda; Arthropoda; Mandibulata; Pancrustacea; Hexapoda; Insecta; Dicondylia; Pterygota; Neoptera; Endopterygota; Amphiesmenoptera; Lepidoptera; Glossata; Neolepidoptera; Heteroneura; Ditrysia; Gelechioidea; Coleophoridae; *Coleophora*; *Coleophora deauratella* Zeller, 1846 (NCBI:txid687026).

## Background

The Red-clover Case-bearer *Coleophora deauratella* is a micromoth in the family Coleophoridae. It is a small moth with a wingspan of 11 mm and shiny metallic wings with bronze and copper tones ( Kimber, 2024).

*Coleophora deauratella* was separated from the similar *C. frischella* in 1976, so precise details of its range are uncertain, but it is known to be common in grassy habitats in some areas of Southern England, where adults are on the wing in June and July ( Kimber, 2024).

The larvae feed on the developing seeds of the Red Clover *Trifolium pratense* ( Kimber, 2024). It causes only minor damage in its native range of Europe, the Middle East and Eastern Siberia but it is an invasive pest on Red Clover elsewhere ( Mori & Evenden, 2015). *Coleophora deauratella* has spread through a wide area of the United States and Canada ( Evenden *et al.*, 2010; Mori *et al.*, 2016; Mori & Evenden, 2015) and has colonised New Zealand ( Dorman *et al.*, 2024). The two main methods of control used are synthetic pheromones to disrupt mating and biological controls using parasitoids ( Kaur *et al.*, 2024).

We present a chromosomal-level genome sequence for a female *Coleophora deauratella*, sequenced as part of the Darwin Tree of Life Project, a collaborative effort to sequence all named eukaryotic species in the Atlantic Archipelago of Britain and Ireland.

## Genome sequence report

**Figure 1.**
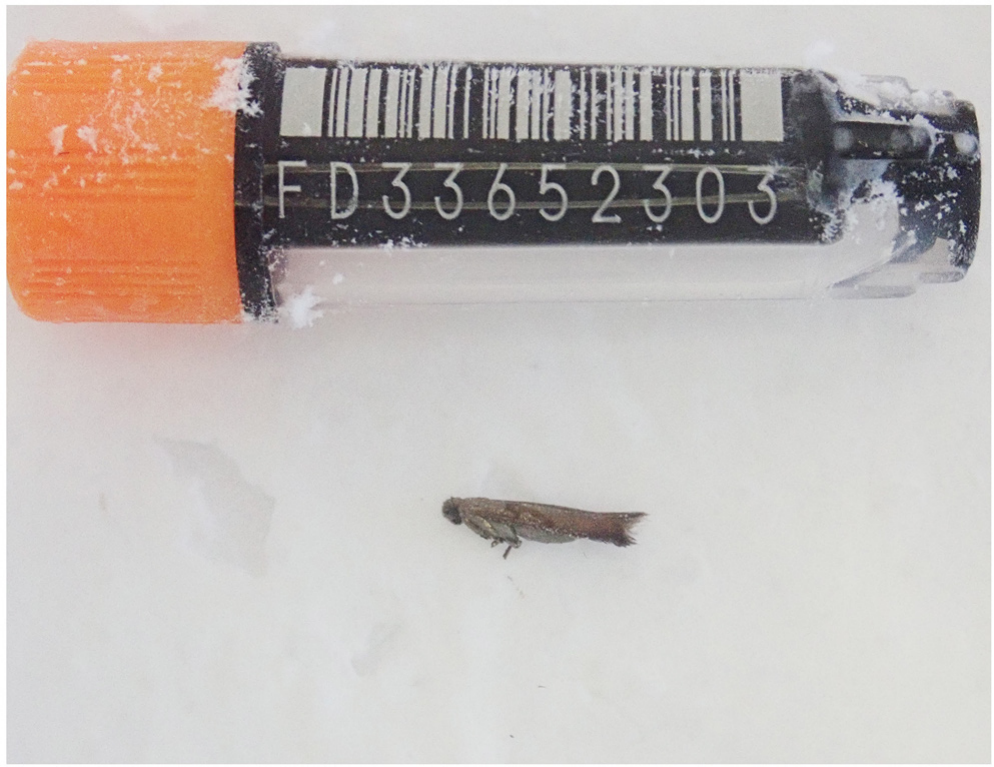
Photograph of the *Coleophora deauratella* (ilColDeau1) specimen used for genome sequencing.

The genome was sequenced from an adult female *Coleophora deauratella* ( Figure 1) collected from Trap Grounds, Oxfordshire, UK (51.77, –1.27). A total of 54-fold coverage in Pacific Biosciences single-molecule HiFi long reads was generated. Primary assembly contigs were scaffolded with chromosome conformation Hi-C data. Manual assembly curation corrected 18 missing joins or mis-joins and removed 6 haplotypic duplications, reducing the assembly length by 0.66% and the scaffold number by 12.82%, and increasing the scaffold N50 by 0.25%.

**Figure 2.**
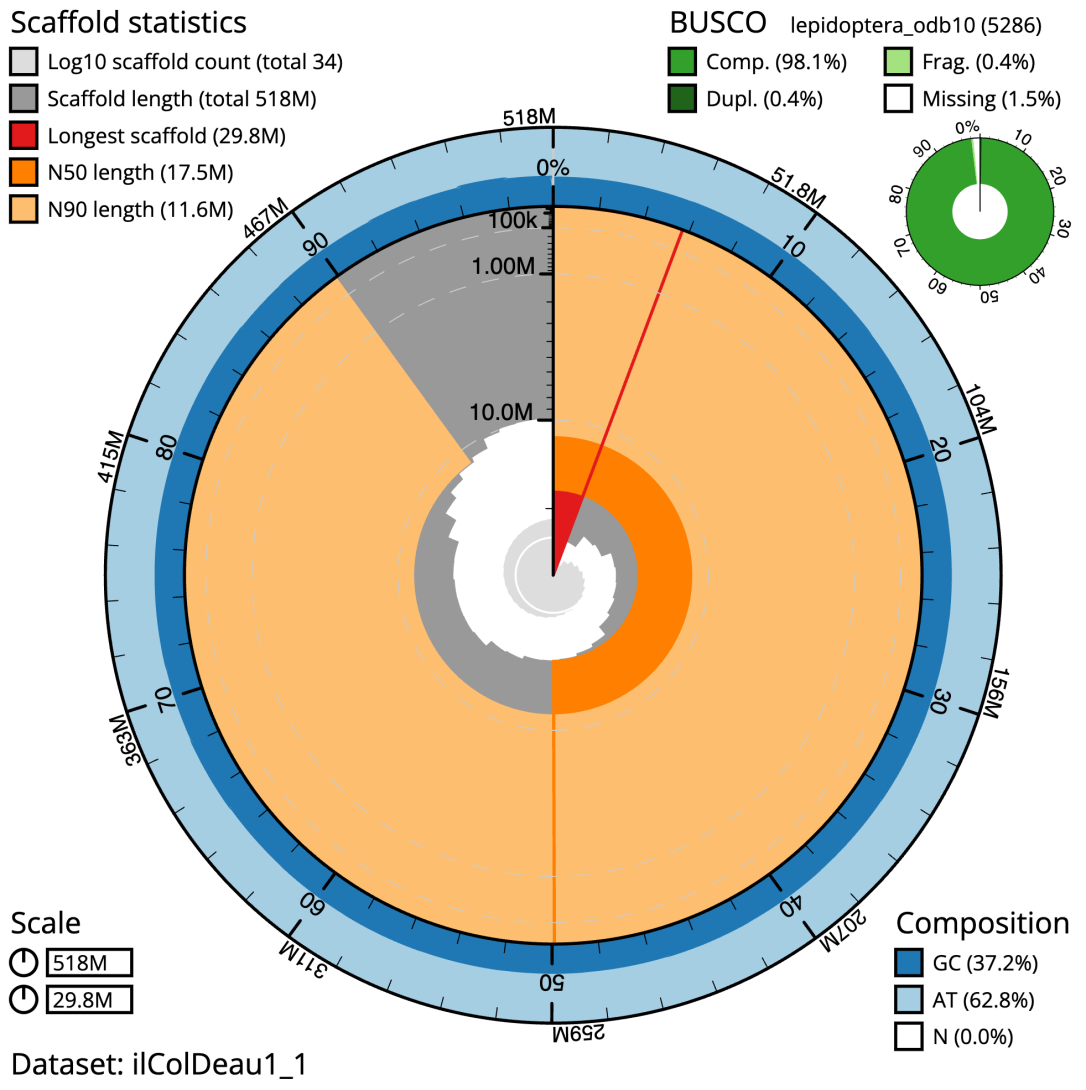
Genome assembly of *Coleophora deauratella*, ilColDeau1.1: metrics. The BlobToolKit snail plot shows N50 metrics and BUSCO gene completeness. The main plot is divided into 1,000 size-ordered bins around the circumference with each bin representing 0.1% of the 518,461,959 bp assembly. The distribution of scaffold lengths is shown in dark grey with the plot radius scaled to the longest scaffold present in the assembly (29,780,019 bp, shown in red). Orange and pale-orange arcs show the N50 and N90 scaffold lengths (17,488,061 and 11,553,269 bp), respectively. The pale grey spiral shows the cumulative scaffold count on a log scale with white scale lines showing successive orders of magnitude. The blue and pale-blue area around the outside of the plot shows the distribution of GC, AT and N percentages in the same bins as the inner plot. A summary of complete, fragmented, duplicated and missing BUSCO genes in the lepidoptera_odb10 set is shown in the top right. An interactive version of this figure is available at https://blobtoolkit.genomehubs.org/view/Coleophora%20deauratella/dataset/ilColDeau1_1/snail.

**Figure 3.**
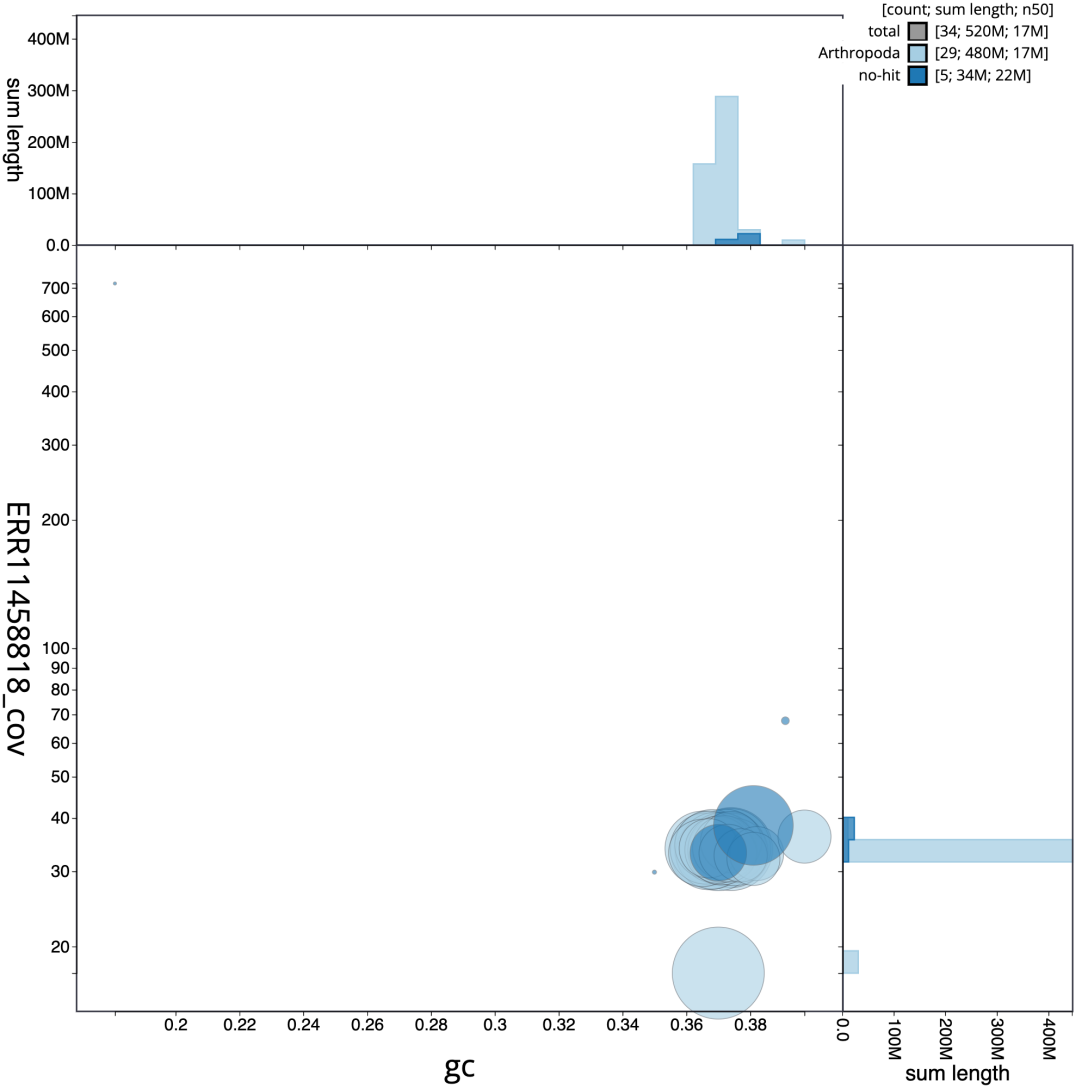
Genome assembly of *Coleophora deauratella*, ilColDeau1.1: BlobToolKit GC-coverage plot. Sequences are coloured by phylum. Circles are sized in proportion to sequence length. Histograms show the distribution of sequence length sum along each axis. An interactive version of this figure is available at https://blobtoolkit.genomehubs.org/view/Coleophora%20deauratella/dataset/ilColDeau1_1/blob.

**Figure 4.**
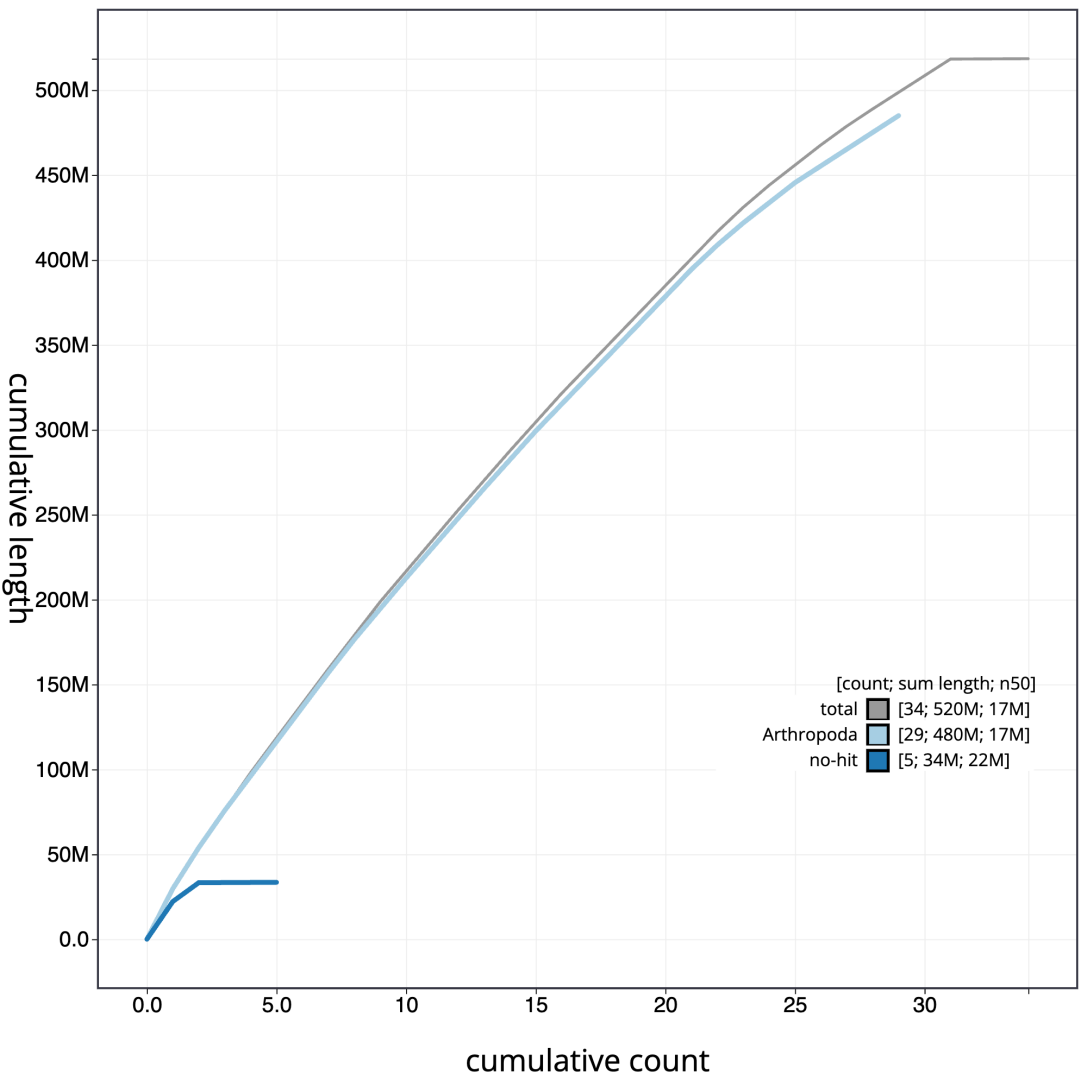
Genome assembly of *Coleophora deauratella* ilColDeau1.1: BlobToolKit cumulative sequence plot. The grey line shows cumulative length for all sequences. Coloured lines show cumulative lengths of sequences assigned to each phylum using the buscogenes taxrule. An interactive version of this figure is available at https://blobtoolkit.genomehubs.org/view/Coleophora%20deauratella/dataset/ilColDeau1_1/cumulative.

**Figure 5.**
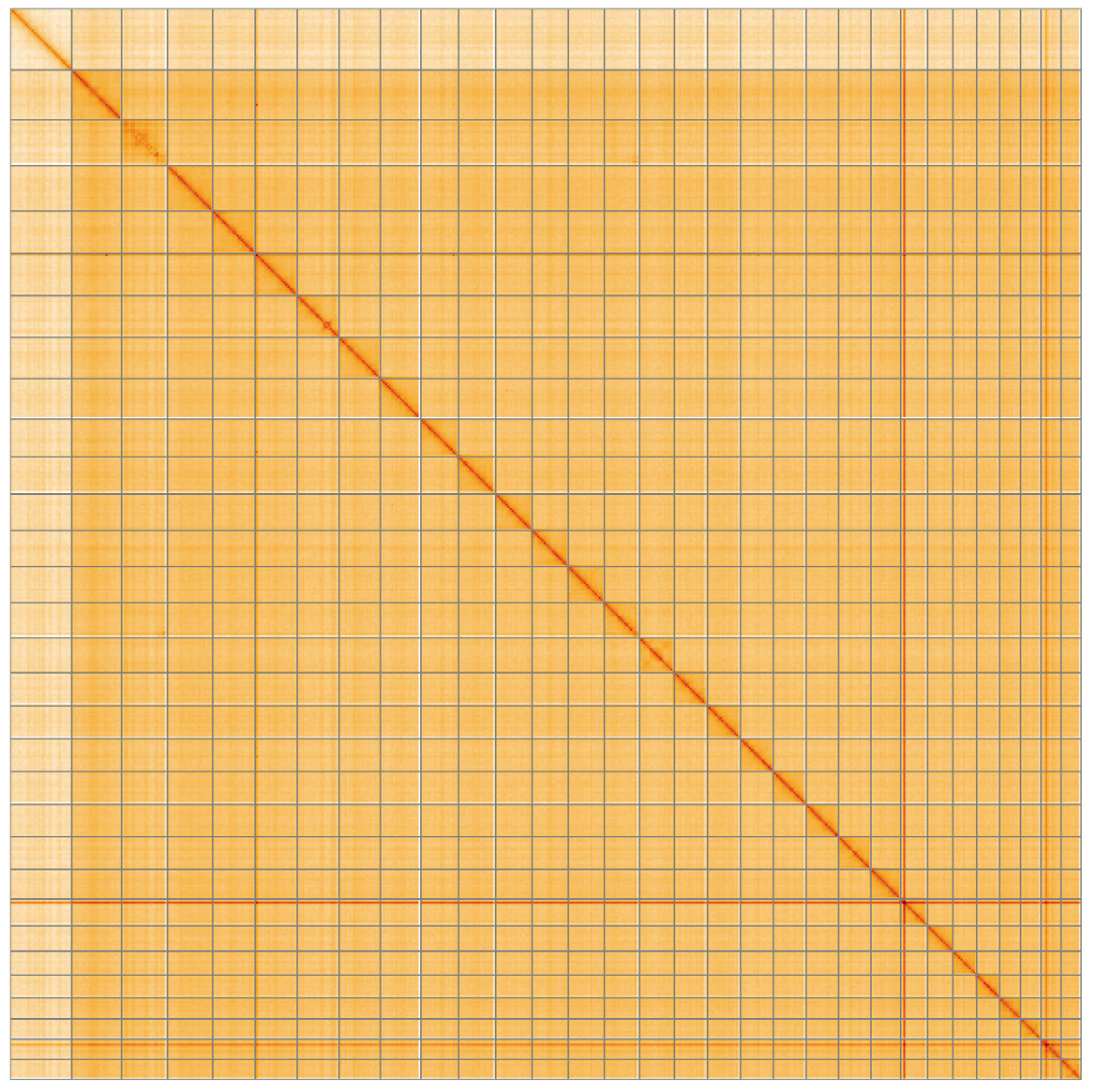
Genome assembly of *Coleophora deauratella* ilColDeau1.1: Hi-C contact map of the ilColDeau1.1 assembly, visualised using HiGlass. Chromosomes are shown in order of size from left to right and top to bottom. An interactive version of this figure may be viewed at https://genome-note-higlass.tol.sanger.ac.uk/l/?d=ceYHNPkBQWyEFd5eAAdDGQ.

The final assembly has a total length of 518.4 Mb in 33 sequence scaffolds with a scaffold N50 of 17.5 Mb ( Table 1). The snail plot in Figure 2 provides a summary of the assembly statistics, while the distribution of assembly scaffolds on GC proportion and coverage is shown in Figure 3. The cumulative assembly plot in Figure 4 shows curves for subsets of scaffolds assigned to different phyla. Most (99.99%) of the assembly sequence was assigned to 31 chromosomal-level scaffolds, representing 29 autosomes and the Z and W sex chromosomes. Chromosome-scale scaffolds confirmed by the Hi-C data are named in order of size ( Figure 5; Table 2). The Z and W chromosomes were assigned based on synteny to *Coleophora flavipennella* (GCA_947284805.1) ( Boyes *et al.*, 2024). While not fully phased, the assembly deposited is of one haplotype. Contigs corresponding to the second haplotype have also been deposited. The mitochondrial genome was also assembled and can be found as a contig within the multifasta file of the genome submission.

**Table 1.** Genome data for *Coleophora deauratella*, ilColDeau1.1.

Project accession data		
Assembly identifier	ilColDeau1.1	
Species	*Coleophora deauratella*	
Specimen	ilColDeau1	
NCBI taxonomy ID	687026	
BioProject	PRJEB62177	
BioSample ID	PacBio and Hi-C sequencing: SAMEA112232927	
Isolate information	ilColDeau1: whole organism (PacBio long read sequencing and Hi-C sequencing)	
Assembly metrics *		*Benchmark*	
Consensus quality (QV)	66.6	*≥ 50*
*k*-mer completeness	100.0%	*≥ 95%*
BUSCO **	C:98.1%[S:97.7%,D:0.4%],F:0.4%, M:1.5%,n:5,286	*C ≥ 95%*
Percentage of assembly mapped to chromosomes	99.99%	*≥ 95%*
Sex chromosomes	ZW	*localised homologous pairs*
Organelles	Mitochondrial genome: 15.76 kb	*complete single alleles*
Raw data accessions		
PacificBiosciences Sequel IIe	ERR11458818	
Hi-C Illumina	ERR11468747	
Genome assembly		
Assembly accession	GCA_958295455.1	
*Accession of alternate haplotype*	GCA_958295505.1	
Span (Mb)	518.4	
Number of contigs	96	
Contig N50 length (Mb)	10.0	
Number of scaffolds	33	
Scaffold N50 length (Mb)	17.5	
Longest scaffold (Mb)	29.78	

* Assembly metric benchmarks are adapted from column VGP-2020 of “Table 1: Proposed standards and metrics for defining genome assembly quality” from Rhie *et al.* (2021). ** BUSCO scores based on the lepidoptera_odb10 BUSCO set using version 5.3.2. C = complete [S = single copy, D = duplicated], F = fragmented, M = missing, n = number of orthologues in comparison. A full set of BUSCO scores is available at https://blobtoolkit.genomehubs.org/view/Coleophora%20deauratella/dataset/ilColDeau1_1/busco.

**Table 2.** Chromosomal pseudomolecules in the genome assembly of *Coleophora deauratella*, ilColDeau1.

INSDC accession	Name	Length (Mb)	GC%
OY282388.1	1	24.12	37.5
OY282390.1	2	21.88	37.5
OY282391.1	3	20.52	37.0
OY282392.1	4	20.38	37.0
OY282393.1	5	20.26	36.5
OY282394.1	6	19.99	37.0
OY282395.1	7	19.56	36.5
OY282396.1	8	18.29	37.0
OY282397.1	9	17.94	37.0
OY282398.1	10	17.64	36.5
OY282399.1	11	17.49	37.0
OY282400.1	12	17.44	37.0
OY282401.1	13	17.02	37.5
OY282402.1	14	16.88	37.0
OY282403.1	15	16.04	36.5
OY282404.1	16	15.91	37.5
OY282405.1	17	15.89	36.5
OY282406.1	18	15.86	37.0
OY282407.1	19	15.72	37.0
OY282408.1	20	15.68	37.0
OY282409.1	21	14.39	37.0
OY282410.1	22	13.02	37.5
OY282411.1	23	12.21	36.5
OY282412.1	24	11.55	37.5
OY282413.1	25	11.09	37.0
OY282414.1	26	10.12	38.0
OY282415.1	27	9.77	39.5
OY282416.1	28	9.83	37.5
OY282417.1	29	9.76	38.0
OY282389.1	W	22.21	38.0
OY282387.1	Z	29.78	37.0
OY282418.1	MT	0.02	18.0

The estimated Quality Value (QV) of the final assembly is 66.6 with *k*-mer completeness of 100.0%, and the assembly has a BUSCO v5.3.2 completeness of 98.1% (single = 97.7%, duplicated = 0.4%), using the lepidoptera_odb10 reference set ( *n* = 5,286).

Metadata for specimens, barcode results, spectra estimates, sequencing runs, contaminants and pre-curation assembly statistics are given at https://links.tol.sanger.ac.uk/species/687026.

## Methods

### Sample acquisition and nucleic acid extraction

A female adult *Coleophora deauratella* (specimen ID Ox002247, ToLID ilColDeau1) was collected from Trap Grounds, Oxfordshire, UK (latitude 51.77, longitude –1.27) on 2022-06-07 by potting. The specimen was collected and identified by Liam Crowley (University of Oxford) and preserved on dry ice.

The workflow for high molecular weight (HMW) DNA extraction at the Wellcome Sanger Institute (WSI) Tree of Life Core Laboratory includes a sequence of core procedures: sample preparation; sample homogenisation, DNA extraction, fragmentation, and clean-up. In sample preparation, the ilColDeau1 sample was weighed and dissected on dry ice ( Jay *et al.*, 2023). Tissue from the whole organism was homogenised using a PowerMasher II tissue disruptor ( Denton *et al.*, 2023a). HMW DNA was extracted in the WSI Scientific Operations core using the Automated MagAttract v2 protocol ( Oatley *et al.*, 2023). The DNA was sheared into an average fragment size of 12–20 kb in a Megaruptor 3 system with speed setting 31 ( Bates *et al.*, 2023). Sheared DNA was purified by solid-phase reversible immobilisation ( Strickland *et al.*, 2023): in brief, the method employs a 1.8X ratio of AMPure PB beads to sample to eliminate shorter fragments and concentrate the DNA. The concentration of the sheared and purified DNA was assessed using a Nanodrop spectrophotometer and Qubit Fluorometer and Qubit dsDNA High Sensitivity Assay kit. Fragment size distribution was evaluated by running the sample on the FemtoPulse system.

Protocols developed by the WSI Tree of Life laboratory are publicly available on protocols.io ( Denton *et al.*, 2023b).

### Sequencing

Pacific Biosciences HiFi circular consensus DNA sequencing libraries were constructed according to the manufacturers’ instructions. Poly(A) RNA-Seq libraries were constructed using the NEB Ultra II RNA Library Prep kit. DNA and RNA sequencing was performed by the Scientific Operations core at the WSI on a Pacific Biosciences Sequel IIe (HiFi) instrument. Hi-C data were also generated from remaining whole organism tissue of ilColDeau1 using the Arima v2 kit. The Hi-C sequencing was performed using paired-end sequencing with a read length of 150 bp on the Illumina NovaSeq 6000 instrument.

### Genome assembly and curation

Assembly was carried out with Hifiasm ( Cheng *et al.*, 2021) and haplotypic duplication was identified and removed with purge_dups ( Guan *et al.*, 2020). The assembly was then scaffolded with Hi-C data ( Rao *et al.*, 2014) using YaHS ( Zhou *et al.*, 2023). The assembly was checked for contamination and corrected as described previously ( Howe *et al.*, 2021). Manual curation was performed using HiGlass ( Kerpedjiev *et al.*, 2018) and PretextView ( Harry, 2022). The mitochondrial genome was assembled using MitoHiFi ( Uliano-Silva *et al.*, 2023), which runs MitoFinder ( Allio *et al.*, 2020) or MITOS ( Bernt *et al.*, 2013) and uses these annotations to select the final mitochondrial contig and to ensure the general quality of the sequence.

### Evaluation of final assembly

A Hi-C map for the final assembly was produced using bwa-mem2 ( Vasimuddin *et al.*, 2019) in the Cooler file format ( Abdennur & Mirny, 2020). To assess the assembly metrics, the *k*-mer completeness and QVy consensus quality values were calculated in Merqury ( Rhie *et al.*, 2020). This work was done using Nextflow ( Di Tommaso *et al.*, 2017) DSL2 pipelines “sanger-tol/readmapping” ( Surana *et al.*, 2023a) and “sanger-tol/genomenote” ( Surana *et al.*, 2023b). The genome was analysed within the BlobToolKit environment ( Challis *et al.*, 2020) and BUSCO scores ( Manni *et al.*, 2021; Simão *et al.*, 2015) were calculated.

Table 3 contains a list of relevant software tool versions and sources.

**Table 3.** Software tools: versions and sources.

Software tool	Version	Source
BlobToolKit	5.3.2	https://github.com/blobtoolkit/blobtoolkit
BUSCO	4.2.1	https://gitlab.com/ezlab/busco
Hifiasm	0.16.1-r375	https://github.com/chhylp123/hifiasm
HiGlass	1.11.6	https://github.com/higlass/higlass
Merqury	MerquryFK	https://github.com/thegenemyers/MERQURY.FK
MitoHiFi	2	https://github.com/marcelauliano/MitoHiFi
PretextView	0.2	https://github.com/sanger-tol/PretextView
purge_dups	1.2.3	https://github.com/dfguan/purge_dups
sanger-tol/genomenote	v1.0	https://github.com/sanger-tol/genomenote
sanger-tol/readmapping	1.1.0	https://github.com/sanger-tol/readmapping/tree/1.1.0
YaHS	yahs-1.1.91eebc2	https://github.com/c-zhou/yahs

### Wellcome Sanger Institute – Legal and Governance

The materials that have contributed to this genome note have been supplied by a Darwin Tree of Life Partner. The submission of materials by a Darwin Tree of Life Partner is subject to the **‘Darwin Tree of Life Project Sampling Code of Practice’**, which can be found in full on the Darwin Tree of Life website here. By agreeing with and signing up to the Sampling Code of Practice, the Darwin Tree of Life Partner agrees they will meet the legal and ethical requirements and standards set out within this document in respect of all samples acquired for, and supplied to, the Darwin Tree of Life Project. 

Further, the Wellcome Sanger Institute employs a process whereby due diligence is carried out proportionate to the nature of the materials themselves, and the circumstances under which they have been/are to be collected and provided for use. The purpose of this is to address and mitigate any potential legal and/or ethical implications of receipt and use of the materials as part of the research project, and to ensure that in doing so we align with best practice wherever possible. The overarching areas of consideration are:

• Ethical review of provenance and sourcing of the material

• Legality of collection, transfer and use (national and international) 

Each transfer of samples is further undertaken according to a Research Collaboration Agreement or Material Transfer Agreement entered into by the Darwin Tree of Life Partner, Genome Research Limited (operating as the Wellcome Sanger Institute), and in some circumstances other Darwin Tree of Life collaborators.

## Data Availability

European Nucleotide Archive: *Coleophora deauratella* (red-clover case-bearer). Accession number PRJEB62177; https://identifiers.org/ena.embl/PRJEB62177 ( Wellcome Sanger Institute, 2023). The genome sequence is released openly for reuse. The *Coleophora deauratella* genome sequencing initiative is part of the Darwin Tree of Life (DToL) project. All raw sequence data and the assembly have been deposited in INSDC databases. The genome will be annotated using available RNA-Seq data and presented through the Ensembl pipeline at the European Bioinformatics Institute. Raw data and assembly accession identifiers are reported in Table 1.

## Author information

Members of the University of Oxford and Wytham Woods Genome Acquisition Lab are listed here: https://doi.org/10.5281/zenodo.7125292.

Members of the Darwin Tree of Life Barcoding collective are listed here: https://doi.org/10.5281/zenodo.4893703.

Members of the Wellcome Sanger Institute Tree of Life Management, Samples and Laboratory team are listed here: https://doi.org/10.5281/zenodo.10066175.

Members of Wellcome Sanger Institute Scientific Operations: Sequencing Operations are listed here: https://doi.org/10.5281/zenodo.10043364.

Members of the Wellcome Sanger Institute Tree of Life Core Informatics team are listed here: https://doi.org/10.5281/zenodo.10066637.

Members of the Tree of Life Core Informatics collective are listed here: https://doi.org/10.5281/zenodo.5013541.

Members of the Darwin Tree of Life Consortium are listed here: https://doi.org/10.5281/zenodo.4783558.
