# Supplementary material for: The genome sequence of the Red-clover Case-bearer, Coleophora deauratella Zeller, 1846
Source: Wellcome Open Res. 2024 Jul 10;9:370. [Version 1] doi: 10.12688/wellcomeopenres.22581.1 (PMC11513577; doi:10.12688/wellcomeopenres.22581.1)
Supplement: Peer review history

# Reviewer report: Reviewer response for version 1

**DOI:** 10.21956/wellcomeopenres.24879.r97112

**Published:** 2024-10-27

**Authors:** Théo Léger

**Affiliations:** 1, Leibniz-Institut für Evolutions- und Biodiversitaetsforschung, Museum für Naturkunde Berlin, Berlin, Germany

The authors present the first complete genome sequence of Coleophora deauratella Zeller, 1846. General information about the species is presented in the introduction. Regarding the host plant, the species is also reported feeding on T. hybridum, T. medium and T. ochroleucon (Baldizzone, 2019; see reference added below).

 Although not being very versed in genome analyses, the value of the scaffold N50 and completeness of the BUSCO genes retrieved indicate high quality genome.

 I would thus accept the paper in the present state. Adding the information about the larval host plant would be appreciated.

 Théo Léger

Are sufficient details of methods and materials provided to allow replication by others?

Yes

Is the rationale for creating the dataset(s) clearly described?

Yes

Are the datasets clearly presented in a useable and accessible format?

Yes

Are the protocols appropriate and is the work technically sound?

Yes

Reviewer Expertise:

systematics, taxonomy, phylogenomics

I confirm that I have read this submission and believe that I have an appropriate level of expertise to confirm that it is of an acceptable scientific standard.

## References

[1] Baldizzone, G. (2019): Fauna d’Italia. Vol. LIII. Lepidoptera Coleophoridae. - XVII + 907 S.; Milano (Calderini).

# Reviewer report: Reviewer response for version 1

**DOI:** 10.21956/wellcomeopenres.24879.r90936

**Published:** 2024-08-09

**Authors:** Sivasankaran Kuppusamy

**Affiliations:** 1 Entomology Research Institute, Loyola College, Chennai, India

The authors have assembled and annotated of *Coleophora deauratella* Zeller, 1846 genomic sequence. They sequenced about 518.4 mega bases of the assembled genome using 33 scaffolds. Standard techniques have been employed to isolate DNA, and the relevant software has been used for sequence assembly and annotation.

**Comments on the manuscript:**

1. The authors have constantly given the complete form of the genus name *Coleophora deauratella* in the article. The genus name may be presented in full the first time, and then in abbreviated form later on, such as *C. deauratella*
2. Author’s name of the species should be included in the text also. The first line of the background can be modified as “The Red-clover Case-bearer *Coleophora deauratella* Zeller, 1846……”
3. “collected from Trap Grounds Oxfordshire, UK (51.77, –1.27)” can be removed from the first sentence of the Genome sequence report. The collection details of the species need not be mentioned here.
4. The mitochondrial genome was annotated but the exact number of mitochondrial genome sequence lengths was not mentioned in the text. The mitogenome sequence length could be given in the text.
5. Total number of PCGs (Protein-coding genes) and number of gene transcripts aren’t given in table 1. Those details should be included in the table.

Above all, I confirm that the manuscript meets the necessary scientific standard and is suitable for indexing"

Are sufficient details of methods and materials provided to allow replication by others?

Yes

Is the rationale for creating the dataset(s) clearly described?

Yes

Are the datasets clearly presented in a useable and accessible format?

Yes

Are the protocols appropriate and is the work technically sound?

Yes

Reviewer Expertise:

Phylogenetic analysis of Noctuoidea moths using NGS techniques

I confirm that I have read this submission and believe that I have an appropriate level of expertise to confirm that it is of an acceptable scientific standard.
